# Dosimetric verification of compensated beams using radiographic film

**DOI:** 10.2478/v10019-011-0020-9

**Published:** 2011-07-20

**Authors:** Slaven Jurkovic, Gordana Zauhar, Dario Faj, Deni Smilovic Radojcic, Manda Svabic, Mladen Kasabasic, Ana Diklic

**Affiliations:** 1 University Hospital Rijeka, Radiotherapy Department, Physics Division, Rijeka, Croatia; 2 School of Medicine, Department of Physics, Rijeka, Croatia; 3 University Hospital Osijek, Department of Radiotherapy and Oncology, Osijek, Croatia

**Keywords:** radiation therapy, dosimetry, compensators

## Abstract

**Introduction:**

External photon beam modulation using compensators in order to achieve a desired dose distribution when brachytherapy treatment is followed by external beam radiation is a well-established technique. A compensator modulates the central part of the beam, and the dose beneath the thickest part of the compensator is delivered mostly by scattered, low energy photons. A two-dimensional detector with a good spatial resolution is needed for the verification of those beams. In this work, the influence of different types of detectors on the measured modulated dose distributions was examined.

**Materials and methods:**

Dosimetric verification was performed using X-Omat V, Eastman Kodak radiographic films at different depths in a solid water phantom. The film measurements were compared with those made by ionization chambers. Photon beams were also modelled using EGSnrc Monte Carlo algorithm to explain the measured results.

**Results:**

Monte Carlo calculated over-response of the film under the thickest part of the compensator was over 15%, which was confirmed by measurements. The magnitude of over-response could be associated with changes in the spectra of photon energy in the beam.

**Conclusions:**

The radiographic film can be used for the dosimetry of compensated high energy photon beams, with limitations in volumes where photon spectra are hardly degraded.

## Introduction

Intracavitary application of brachytherapy sources followed by external beam radiation is a common practice in radiotherapy of carcinoma of the cervix. Since the application of brachytherapy sources results in characteristic dose distributions, modulated external photon beams should be added in a way to achieve the desired cumulative dose distribution over the target volume. Several techniques used in practice have been described.^1^–^4^

On the other hand, the dosimetry of modulated linear accelerator’s photon beams is rather complex, mainly due to dose distribution in homogeneity within the radiation field with large dose gradients. Therefore, dosimetric verification needs a high spatial resolution and this demand makes the radiographic film a dosimeter of choice.^5^–^8^ Nevertheless, it has been shown that the absorbed dose could be related to the energy absorption coefficient.^9^ Furthermore, energy absorption coefficients for film emulsions and water differ significantly in the low energy region bellow 400 keV, as the data calculated according to Seltzer have shown.^10^ Since there is an enhanced contribution of scattered radiation to the total dose in modulated photon beams, a disadvantage of the film which shows over-response to low energy photons may become important.^11^–^13^

In this work, we compared the measured dose distributions of high energy photon beams acquired by different detectors. In order to discuss experimental results, Monte Carlo simulation of particle transport for the measured beams was done.

## Materials and methods

Dosimetric verification of open and modulated 6 MV photon beams from Siemens Oncor Impression linear accelerator was performed using X-Omat V (Eastman Kodak) radiographic films at different depths in solid water (PTW Solid Water Phantom). We used fixed source-to-surface (SSD) geometry with SSD=100 cm on the phantom surface. Film dosimetry was performed using Vidar DosimetryPro Advantage scanner with Coherence Physicist (Siemens Medical Solutions) and PIPSPro (Standard Imaging) software packages for film dosimetry. The dose profiles measured by the film were compared with those made by ionization chambers (IBA Dosimetry, compact chambers CC13 and CC 01) in the water phantom (IBA Dosimetry, Blue Phantom). Regarding a better spatial resolution of a small volume ionisation chamber (CC01), data measured with those chambers in the high gradient region of the beams were superimposed on measurements with CC13 ionisation chamber which had a better signal-to-noise ratio.

**FIGURE 1:**
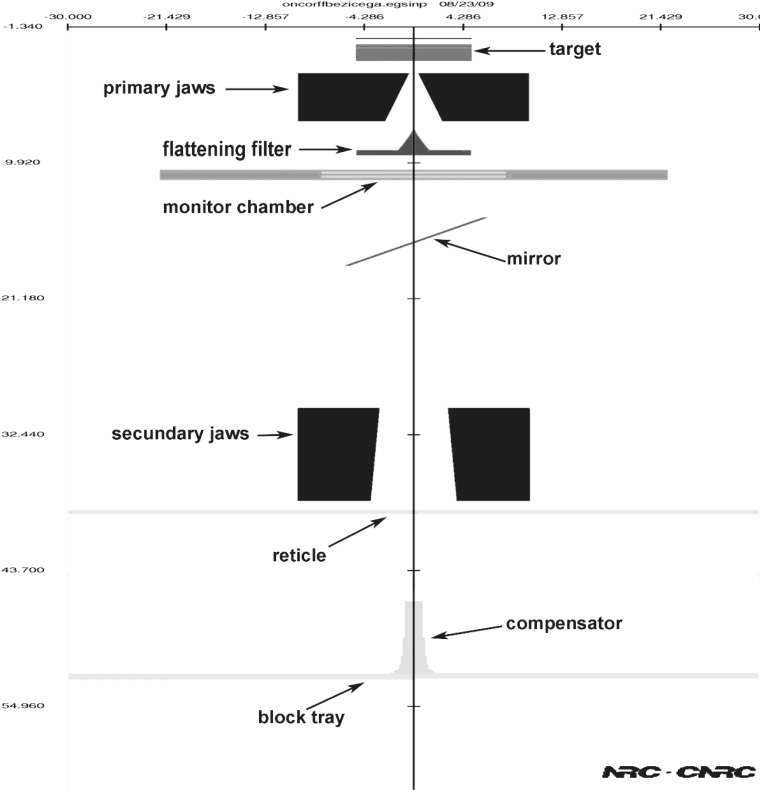
Simulated geometry of accelerator’s head according to manufacturer’s data for modelling compensated beam using BEAMnrc program package.

**FIGURE 2:**
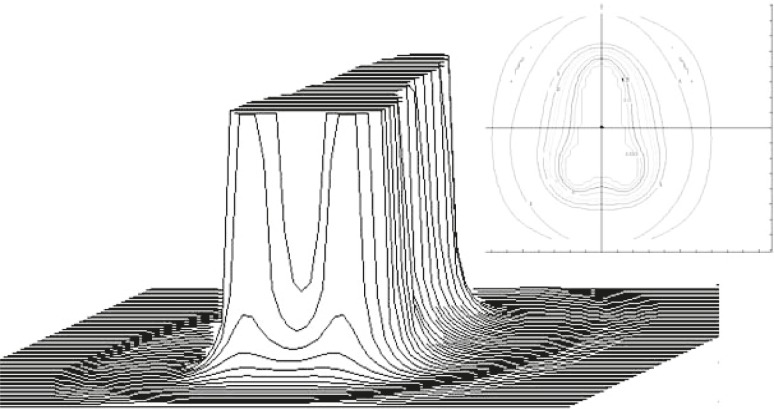
Compensator’s shape calculated to conform the dose distribution given by an external beam according to the dose distribution around brachytherapy sources. The insert shows thickness of the compensator in a form of level curves in mm.

Photon spectra for Siemens Oncor Impression linear accelerator photon beams were calculated at the measuring planes using Monte Carlo simulation of particle transport (EGSnrc). The simulation for 6 MV photons with a field size of 20×20cm^2^ (defined at SSD=100 cm) was performed using OMEGA/BEAM code, developed by the National Research Council of Canada (NRCC). This is an EGSnrc user code capable of complex linear accelerator geometric coding.^14^ The detailed geometry and composition of each individual device in the Siemens Oncor Impression linear accelerator were obtained from the manufacturer. Open and modulated beams were modelled using BEAMnrc software. The modelled geometry of compensated beam is shown in Figure 1. Calculated shape of the compensator is shown in Figure 2.

The compensator shape was calculated using the dose distribution around the brachytherapy sources as a pattern according to which the open photon beam was modulated in order to achieve the desired total dose distribution.^4^

Therefore, the shape of the dose distribution is rather characteristic and, from the dose profiles point of view, three different areas can be distinguished: the area under the compensator and the open beam area where the measurements can be performed with high reproducibility and the area near the edge of the compensator which is characterized by high dose gradients and a lower level of measurement reproducibility. In the last area, Monte Carlo calculation is especially used as a guideline for the interpretation of the measured dose distributions. On the other hand, changes in the energy spectrum were expected in the area under the thickest part of the compensator.

The absorbed dose in a material depends on energy absorption coefficients^9^ and there is a large difference in those coefficients for film and water in low energy area.^10^ Therefore, an over-response of the film under the thickest part of the compensator was expected at larger depths because the Compton scattered low-energy photons dominate there.^8^ Dose calculations in a material were performed according to:^9^

[1] $$D=-\frac{1}{\rho }\nabla \Psi =\int \Psi_{E}=\frac{\mu_{\mathrm{en}}\left(E\right)}{\rho }\mathrm{dE}=\Psi \frac{\bar{\mu_{\mathrm{en}}}}{\rho }$$

The BEAM code was implemented using variance reduction techniques: photon forcing, bremsstrahlung splitting and range rejection to speed up the simulation. The lower charged particle cutoff energy, AE, was 0.7 MeV, and the lower photon cutoff energy, AP, was 0.01 MeV. The energy loss per transport step of the electron, ESTEPE, was controlled by PRESTA.^15^ Scored plane was set at *Z*=100cm to collect the particles after transportation from the accelerator, and to form the phase space file. Information concerning particles in the phase space file included the position (*X*, *Y*, *Z*), direction (*U*, *V*, *W*), energy, charge, weighting, and origin (LATCH). Five to ten million particles were collected in the scored plane. The phase space file served as the source for the following water phantom simulation using DOSXYZ, an EGSnrc user code for 3D absorbed dose calculation in Cartesian coordinates.^16^ In DOSXYZ, the water phantom size was 40×40×40cm^3^ and the phase space source position was on the water surface (*Z*=0). The origin was at the centre of the radiation field. Voxels with size of 0.5×0.5×0.5 cm^3^ (*X*×*Y*×*Z*) were set at the depth of the maximum dose for dose profile simulation. 50 voxels from water surface (*Z*=0) with size 2.0×2.0×0.2 cm^3^ and 20 voxels with size 2.0×2.0×0.4 cm^3^ were set along the central axis for central percent depth dose (PDD) simulation. The particles in the phase space file were redistributed and reused to obtain better accuracy in dose calculation.^16^ Physical parameters of original electron beam that may influence the dose profile and central-axis PDD curve are the beam energy, the beam spot size and the distance from the point source.^17^,^18^ These parameters were adjusted to allow dose profiles and percentage depth dose curve to match measured data. Since we calculated changes in beam energies, for the purpose of our work, the accuracy of the beam profiles was not essential. We decided that 3% discrepancy from measurements is acceptable in the high dose region and 20% in the low dose region. Recommended values are 2% and 20% respectively.^19^–^21^

## Results

**FIGURE 3:**
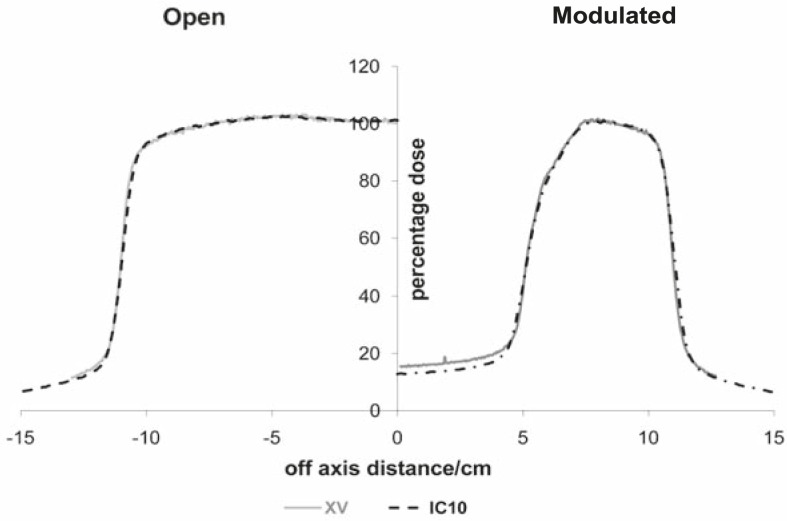
Dose profiles measured with ionization chamber (black) and X-Omat V film (grey). Measurements were done with SSD=100 cm, 20×20 cm2 field size at 10 cm depth for open and modulated beams. Regarding the symmetry of the dose distributions only half-profiles are shown.

From the analysis of measured beam profiles, we observed significant discrepancies between measurements with the radiographic film and ionization chambers when measuring beam profiles of modulated beams on larger depths in water. The discrepancies were pronounced under the thickest part of the compensator (Figure 3).

**FIGURE 4:**
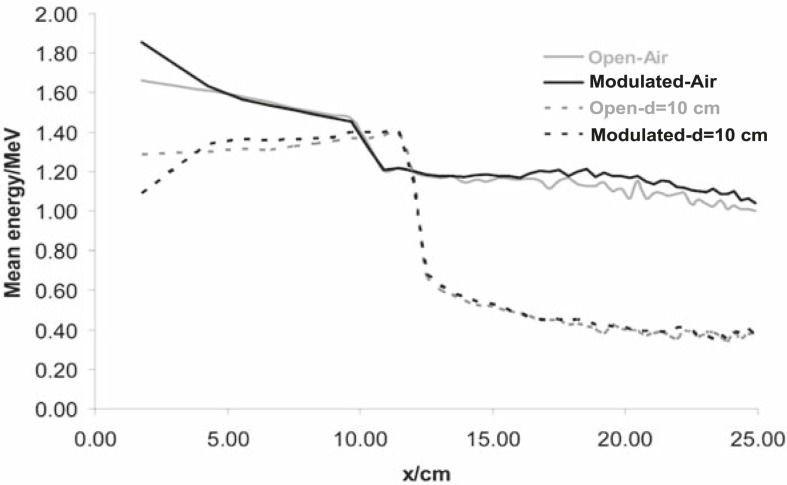
Mean energy distributions for open and modulated beams in air and on 10 cm depth in water. Calculations were done with SSD=100 cm and 20×20 cm2 field size for open and modulated beams. Regarding the symmetry of the energy distributions only half-profiles are shown.

Calculated mean energy distributions in open and modulated beams are shown in Figure 4.

From the ‘in air’ simulation analyses, we can see that the compensator removed low energy photons from the beam, so the mean energy of the modulated beam is higher than the one of the open beam (Figure 4). This can also be seen in Figure 5A. On the other hand, at larger depths in water, the Compton scattering low-energy photons dominate, especially under the thickest part of the compensator, so the Figure 4 shows the decrease of the mean energy of the modulated beam there. Calculated photon spectral distributions for open and modulated beams are shown in Figures 5A and 5B, respectively.

Taking into account the dependence of mass absorption coefficients on photon energy for used dosimeters and calculated energy distribution of photons in small volumes, we can estimate changes in the film response. Regarding data shown in Figures 4, 5A and 5B, it follows that the largest differences could be expected under the thickest part of the compensator at larger depths in water because of the largest energy degradation. Dose calculations for the film and water were done according to Equation [1]. Calculated over-response of the film in this region was over 15% which was confirmed by measurements (Figure 3).

## Discussion

In this paper, we showed that magnitude of over-response of the radiographic film of modulated high energy photon beams could be associated with the changes in the spectra of photon energy in the beam. Since the largest spectral change was under the thickest part of the compensator, there was the largest difference between film and ionisation chamber measurements.

Regarding a high dose gradient beneath the steep part of the compensator, it was not possible to measure doses in this area accurately. Nevertheless, spectra in this area resemble open rather than modulated beam beneath the thickest part of the compensator. In this way, the over-response of the film under the steep part of the compensator would be small.

The radiographic film is often used for verifications of modulated photon beams.^5^,^7^,^8^,^22^ Despite of described limitations, it can be used either on build up depth for the evaluation of compensators shape or for measuring dose distributions of modulated high energy photon beams in phantoms. Special attention should be paid to the interpretation of measured values in volumes where photon spectra are hardly degraded.

## Figures and Tables

**FIGURE 5A:**
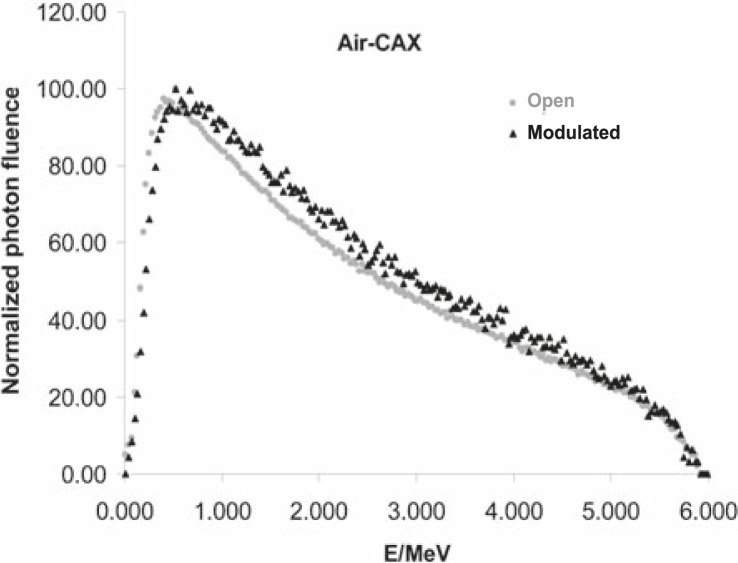
Photon spectral distributions for open and modulated 6MV photon beams on central axis in air. Calculations were done with SSD=100 cm and 20×20 cm2 field size.

**FIGURE 5B:**
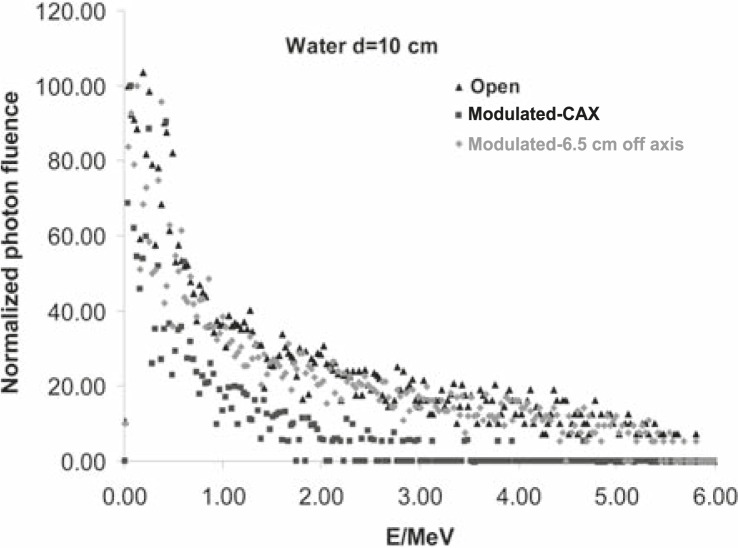
Photon spectral distributions in water for open and modulated 6MV photon beams, on 10 cm depth in water. Calculations were done with SSD=100 cm and 20×20 cm2 field size at central axis for open beam and also at central axis and under steep part of the compensator (6.5 cm off axis) for modulated beam.
